# Spatial analysis of avoidable hospitalizations due to tuberculosis in Ribeirao Preto, SP, Brazil (2006-2012)

**DOI:** 10.1590/S1518-8787.2016050006049

**Published:** 2016-04-27

**Authors:** Mellina Yamamura, Isabela Moreira de Freitas, Marcelino Santo, Francisco Chiaravalloti, Marcela Antunes Paschoal Popolin, Luiz Henrique Arroyo, Ludmila Barbosa Bandeira Rodrigues, Juliane Almeida Crispim, Ricardo Alexandre Arcêncio

**Affiliations:** I Programa de Pós-Graduação em Saúde Pública. Escola de Enfermagem de Ribeirão Preto. Universidade de São Paulo. Ribeirão Preto, SP, Brasil; II Departamento de Enfermagem. Universidade Federal do Maranhão. Imperatriz, MA, Brasil; III Departamento de Epidemiologia. Faculdade de Saúde Pública. Universidade de São Paulo. São Paulo, SP, Brasil; IV Departamento de Medicina. Universidade Federal do Mato Grosso. Sinop, MT, Brasil; V Departamento de Enfermagem Materno Infantil e Saúde Pública. Escola de Enfermagem de Ribeirão Preto. Universidade de São Paulo. Ribeirão Preto, SP, Brasil

**Keywords:** Tuberculosis, epidemiology, Hospitalization, Spatial Analysis, Geographic Information Systems, Ecological Studies

## Abstract

**OBJECTIVE:**

To describe the spatial distribution of avoidable hospitalizations due to tuberculosis in the municipality of Ribeirao Preto, SP, Brazil, and to identify spatial and space-time clusters for the risk of occurrence of these events.

**METHODS:**

This is a descriptive, ecological study that considered the hospitalizations records of the Hospital Information System of residents of Ribeirao Preto, SP, Southeastern Brazil, from 2006 to 2012. Only the cases with recorded addresses were considered for the spatial analyses, and they were also geocoded. We resorted to Kernel density estimation to identify the densest areas, local empirical Bayes rate as the method for smoothing the incidence rates of hospital admissions, and scan statistic for identifying clusters of risk. Softwares ArcGis 10.2, TerraView 4.2.2, and SaTScan^TM^ were used in the analysis.

**RESULTS:**

We identified 169 hospitalizations due to tuberculosis. Most were of men (n = 134; 79.2%), averagely aged 48 years (SD = 16.2). The predominant clinical form was the pulmonary one, which was confirmed through a microscopic examination of expectorated sputum (n = 66; 39.0%). We geocoded 159 cases (94.0%). We observed a non-random spatial distribution of avoidable hospitalizations due to tuberculosis concentrated in the northern and western regions of the municipality. Through the scan statistic, three spatial clusters for risk of hospitalizations due to tuberculosis were identified, one of them in the northern region of the municipality (relative risk [RR] = 3.4; 95%CI 2.7–4,4); the second in the central region, where there is a prison unit (RR = 28.6; 95%CI 22.4–36.6); and the last one in the southern region, and area of protection for hospitalizations (RR = 0.2; 95%CI 0.2–0.3). We did not identify any space-time clusters.

**CONCLUSIONS:**

The investigation showed priority areas for the control and surveillance of tuberculosis, as well as the profile of the affected population, which shows important aspects to be considered in terms of management and organization of health care services targeting effectiveness in primary health care.

## RESUMO

**OBJETIVO:**

Descrever a distribuição espacial dos casos de internações evitáveis por tuberculose no município de Ribeirão Preto, SP, e identificar aglomerados espaciais e espaço-temporais de risco para a ocorrência desses eventos.

**MÉTODOS:**

Estudo descritivo e ecológico que considerou os registros de internações no Sistema de Informação Hospitalar dos residentes de Ribeirão Preto, SP, no período de 2006 a 2012. Para as análises espaciais foram considerados somente os casos com endereços registrados, sendo os mesmos geocodificados. Recorreu-se à estatística de densidade Kernel para identificar as áreas de maior densidade, taxa bayesiana empírica local como método de suavização das taxas de incidência de internações e estatística de varredura para identificação de aglomerados de risco. Para as análises foram utilizados os *softwares* ArcGis 10.2, TerraView 4.2.2 e SaTScan^TM^.

**RESULTADOS:**

Foram identificadas 169 internações por tuberculose. A maioria das internações ocorreu com pessoas do sexo masculino (n = 134; 79,2%) com idade mediana de 48 anos (DP = 16,2). A forma clínica predominante foi a pulmonar, com confirmação por exame microscópico da expectoração (n = 66; 39,0%). Foram geocodificados 159 (94,0%) casos. Observou-se distribuição espacial não aleatória de internações evitáveis por tuberculose, concentradas nas regiões norte e oeste do município. Por meio da estatística de varredura, identificaram-se três aglomerados espaciais de risco para internações por tuberculose, um na região norte do município (risco relativo [RR] = 3,4; IC95% 2,7–4,4); o segundo, na região central, onde há uma unidade prisional (RR = 28,6; IC95% 22,4–36,6); e o último, na região sul, área de proteção para as internações (RR = 0,2; IC95% 0,2–0,3). Não foram identificados aglomerados espaço-temporais.

**CONCLUSÕES:**

A investigação mostrou áreas prioritárias para o controle e vigilância da tuberculose e um perfil de população atingida, evidenciando aspectos importantes a serem considerados em termos de gestão e organização dos serviços de saúde com vistas à efetividade da Atenção Primária à Saúde.

## INTRODUCTION

Controlling and eliminating tuberculosis is still a world health care challenge. According to a report from the World Health Organization (WHO), nine million new cases occurred in the world in 2013; among these, only 6.5 million were notified. Besides that, 1.5 million of them led to the patient’s demise^a^. Brazil figures in the list of 22 countries with the highest numbers of tuberculosis cases, with an estimated incidence of 46.1 cases per 100,000 inhabitants and a mortality rate of 2.2 per 100,000 inhabitants, which does not consider people infected by HIV^a^.

Among the challenges accepted globally in WHO’s post-2015 agenda is the elimination of this disease until 2050 (less than one case per 100,000 inhabitants)^a^. Nonetheless, the obstacle on the way to achieve this goal is inequality in the access and use of worldwide available technologies that support diagnosis and treatment. Among the markers of quality or effectiveness of health care services in the equal access to health care services by the population affected by tuberculosis^13^ are avoidable hospitalizations.

Avoidable hospitalizations are those that would not take place if health care was handled with quality and timely regarding primary health care (PHC)^1,9^. Tuberculosis has figured in the list of hospitalizations acknowledged as avoidable since 2008^b^.

Hospitalizations due to tuberculosis are a problem in Brazil. A study conducted by San Pedro and Oliveira^28^ (2013) showed that, in 2010, Brazil’s hospitalization rate was 7.2 cases per 100,000 inhabitants and, in the state of Sao Paulo, 4.2 cases per 100,00 inhabitants.

Several studies suggest different hypotheses to explain the occurrence of avoidable hospitalizations^2,6,8,10,16,21,23^. According to these studies, although social and cultural factors may be associated with avoidable hospitalizations due to tuberculosis, the services’ ability to solve problems and produce equality, especially in PHC, has been considered an important determinant.

As it brings financial impacts to patients and to health care systems alike, the cost of a hospital treatment exceeds up to a thousandfold the one of outpatient services^22^, and that is therefore less cost-effective as compared to outpatient treatments^3^.

Although avoidable hospitalizations due to tuberculosis have been targets of investigation in Brazilian studies^2,6,8,16,21,23^, this health condition has not been analyzed under a geographical perspective.

The literature has shown that the organization of health care services, especially PHC, is not the same among territories^16^. That leads to the assumption that hospitalizations due to tuberculosis are not geographically distributed in a homogeneous way. That happens because the several organizational and ideological factors that permeate the logics of PHC end up benefiting less privileged groups, for the offer of a minimum package of poor quality actions and human resources^12^. Thus, supposedly there are territories in which such event is more likely to happen than others, which requires a more thorough investigation.

In that sense, the aim of this study was to describe the spatial distribution of avoidable hospitalizations due to tuberculosis in the municipality of Ribeirao Preto, SP, Brazil, and to identify spatial and space-time clusters for the risk of these events occurring.

## METHODS

This is a descriptive, ecological study conducted in the municipality of Ribeirao Preto, SP, Brazil, located at longitude 47º48’24”W and latitude 21º10’42”S in the northeastern region of the state of Sao Paulo, with a population of 604,682 inhabitants.

Ribeirao Preto falls into group two of the Sao Paulo Social Responsibility Index (IPRS). The State Data System Foundation (SEADE) defines this group as municipalities with high wealth levels but unsatisfactory social indicators – especially concerning Ribeirao Preto’s education level average, which was inferior to the state’s^c^. The municipality is considered a priority in the actions for controlling tuberculosis, as it had an incidence of 23 cases per 100,000 inhabitants in 2013 and 19.0% of deaths in the case of new residents, which is well above the state data (7.2%) in 2012^d^ and the limit recommended by WHO (5.0%)^a^.

In this study, we considered the cases of avoidable hospitalizations due to tuberculosis of residents in the municipality of Ribeirao Preto, who figured in the Hospital Information System of the Brazilian Unified Health System (HIS-SUS) of a state hospital from 2006 to 2012. This hospital is the only reference in the municipality for clinical tuberculosis admissions, and the long-term admissions are referred to other services or other municipality. The data were collected between May 10 and June 14, 2013, and the Hospital Admission Consent Forms selected were the ones whose International Classifications of Diseases, version 10 (ICD-10) were from A15.0 to A17.9 in Main ICD item, recognized as avoidable according to the ordinance issued by the Ministry of Health (MH)^b^.

When more than one hospitalization was identified, only the first record was considered. In the exploratory analysis, the variables considered were sex, age (birth date), year of hospitalization, main clinical form or ICD, and type of discharge (condition or reason to be discharged from the hospital).

Measures of position and dispersion were calculated for the age variable, and these were classified to consider the three main age groups (children, adults, and older adults). Absolute and relative frequencies were calculated for all remaining variables.

Records with incomplete addresses were disregarded as they could not be possibly geocoded, and the same was done for the cases of residents in rural areas, as this is a disease that is characteristic of densely populated and urban areas.

At this stage, Street Base Basic ® (from Image®) street layout tool was used in a shapefile under UTM projection – Zone 23S – Datum WGS1984. The addresses were standardized according to the related base, through the use of the TerraView software (version 4.2.2). In a complementary way, for the records outside the cartographic base, we resorted to the Batch Geocode and FindLatitudeandLongitude tools, both free of charge and allowing geocoding of addresses through access to Google Earth.

The Kernel method was used, and it corresponds to a point density analysis, for the identification and representation of areas with higher density of avoidable hospitalizations due to tuberculosis. This method enables exploratory interpolation, which generates a density surface for the identification of denser areas^17^. Considering a 1,000-meter radius, the theme map of the distribution of densities of avoidable hospitalizations due to tuberculosis, according to home addresses, was generated on the ArcGis software (version 10.2).

The crude incidence rate of avoidable hospitalizations due to tuberculosis was calculated considering the number of hospitalizations occurred in the census area as a denominator and the population residing in the respective sector as denominator. We obtained an annual average of avoidable hospital admission rates as a function of the number of years considered in the study multiplied by 100,000. Choropleth maps were built considering crude and empirical local rates, using the ArcGis software (version 10.2).

As the census area is subject to oscillations in its small or empty numbers, we chose to apply empirical Bayes method to reduce distortions^15^. With this method, it is possible to obtain a weighted average between the crude rate of a census area and the rate of the area with the closest neighbors, which is taken as reference. The Terraview software, version 4.2.2 was used for these analyses. Following that, the scatter plot of local empirical Bayes rates was generated on the ArcGis software (version 10.2) and grouped into quintiles.

In the last step, we used the spatial analysis technique called scan statistic to identify clusters of cases of both high-risk admissions and protection for the occurrence of avoidable hospitalizations.

To identify purely spatial clusters, whose distribution is heterogeneous and events should be rare concerning the population, we used the discrete Poisson equation, as well as the following conditions: no geographical overlaying of clusters; maximum cluster size of 50.0% of the exposed population; round-shaped clusters; and 999 replications. To detect the space-time clusters, the same criteria above were considered, with time accuracy in years and clusters of up to 50.0% of the total period, from 2006 to 2012.

At this stage, the avoidable hospitalizations due to tuberculosis were controlled by variables sex and age, and we resorted to the SaTScan^TM^ software^e^. The relative risks of the clusters were estimated with their respective 95% confidence intervals^11^. Chorochromatic maps of clusters of risk were built on the ArcGis software (version 10.2).

The study was approved by the Research Ethics Committee of the Escola de Enfermagem de Ribeirão Preto of the Universidade de São Paulo (CAAE Protocol 09708612.7.0000.5393).

## RESULTS

From 2006 to 2012, 169 hospitalizations due to tuberculosis were identified in a public hospital of reference for tuberculosis care. Most hospitalizations were of men (n = 134; 79.2%), averagely aged 48 years (SD = 16.2), ranging from 6 to 98 years of age.

Table 1 shows the profile of the cases of avoidable hospitalizations due to tuberculosis. Concerning its clinical form (Table 2), pulmonary tuberculosis was predominant (n = 138; 81.6%). However, 38 cases (22.5%) were not mentioned to include bacilloscopic examination.

**Table 1:** Clinical and epidemiological profile of avoidable hospitalizations due to tuberculosis. Ribeirao Preto, SP, Southeastern Brazil, 2006-2012.

Variable	n	%
Age (years)		
0-14	4	2.3
15-64	143	84.6
≥ 65	22	13.0
Sex		
Male	134	79.2
Female	35	20.7
Year of admission		
2006	25	14.7
2007	28	16.5
2008	23	13.6
2009	22	13.0
2010	32	18.9
2011	23	13.6
2012	16	9.4
Type of discharge		
Hospital discharge	142	84.0
Discharge upon request	9	5.3
Permanence due to other factors	3	1.7
Transference to another establishment	8	4.7
Death	7	4.1

**Table 2:** Clinical cases of avoidable hospital admissions selected to the study. Ribeirao Preto, SP, Southeastern Brazil, 2006-2012.

Code*	Definition	n	%
	Pulmonary tuberculosis		
A15.0	Pulmonary tuberculosis, as confirmed through microscopic examination of expectorated sputum, without or without cell culture.	66	39.0
A15.1	Pulmonary tuberculosis, as confirmed only by cell culture	7	4.1
A15.2	Pulmonary tuberculosis, as histologically confirmed	11	6.5
A15.3	Pulmonary tuberculosis, as confirmed through unspecified means	15	8.9
A16.0	Pulmonary tuberculosis with negative bacteriological and histological exams	0	0
A16.1	Pulmonary tuberculosis with no bacteriological or histological exams	1	0.6
A16.2	Pulmonary tuberculosis, with no mention of bacteriological or histological confirmation	38	22.5

Total		138	81.6

	Extrapulmonary tuberculosis		
A15.4/A16.3	Tuberculosis of intrathoracic lymph nodes, with/without mention of bacteriological or histological confirmation	1	0.6
A15.5/A16.4	Tuberculosis of the larynx, trachea, and bronchi, with/without mention of bacteriological or histological confirmation	1	0.6
A15.6/A16.5	Tuberculous pleurisy, with no mention of bacteriological or histological confirmation	4	2.4
A15.7/A16.7	Primary tuberculosis of the airways, with/without mention of bacteriological or histological confirmation	3	1.8
A15.8/A16.8	Other forms of tuberculosis of the airways, with/without mention of bacteriological or histological confirmation	8	4.7
A15.9/A16.9	Unspecified tuberculosis of the airways, with/without mention of bacteriological or histological confirmation	8	4.7
A17.0	Tuberculous meningitis	0	0
A17.1	Meningeal tuberculoma	1	0.6
A17.8	Other tuberculoses of the nervous system	1	0.6
A17.9	Unspecified tuberculosis of the nervous system	4	2.4

Total		31	18.4

* According to the International Classification of Diseases, version 10.

The procedure adopted in the study enabled the geocoding of 159 cases (94.0%). Four cases (2.4%) were excluded as there were no address data in their admission records, and six of them (3.6%) were found to have inconsistencies regarding their addresses, so it was not possible to identify them through Batch Geocode or FindLatitudeandLongitude.

**Figure 1:**
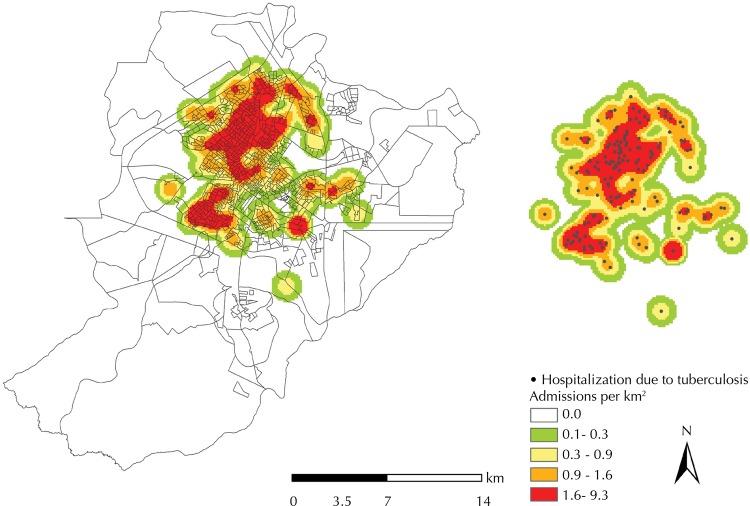
Kernel map of avoidable hospitalizations due to tuberculosis. Ribeirao Preto, SP, Southeastern Brazil, 2006-2012.

The maps generated through the application of the Kernel method (Figure 1) show the locations with the highest densities of cases per square kilometer (km^2^) which are displayed in red. A heterogeneous distribution is observed, with the formation of two possible large clusters, mainly concentrated in the western and northern zones of the municipality. Areas with no admissions were predominant in the municipality’s southern zone.

**Figure 2:**
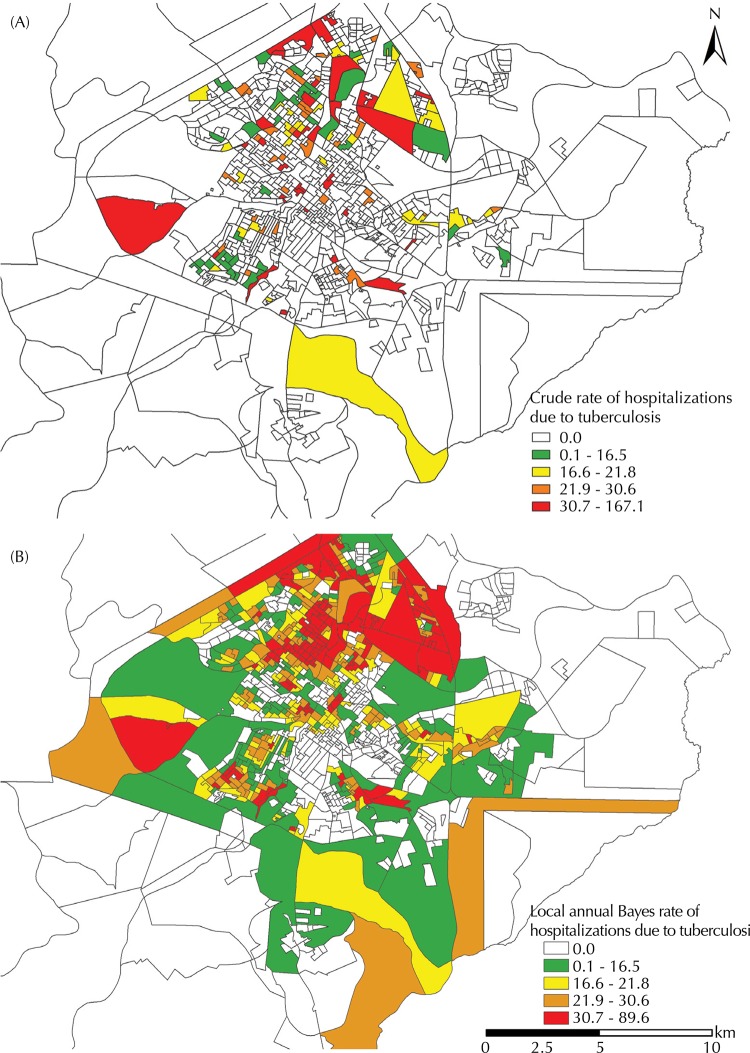
Maps of crude avoidable hospitalizations rates (A) and local empirical Bayes rates (B), both per census areas and in cases per 100,000 inhabitants/year. Ribeirao Preto, SP, Southeastern Brazil, 2006-2012.

Figure 2-A shows the crude avoidable hospitalizations rates and Figure 2-B shows the local empirical Bayes rates per census area. The areas in red in Figure 2-A represent the areas with the highest crude rates of avoidable hospitalizations due to tuberculosis, which ranged from 32 to 167 cases per 100,000 inhabitants.

In part B of Figure 2, it is possible to see, through the empirical Bayes rate, a better straightening of the data, which shows that the census areas with the highest rates, which are shown in red in the map, and also the ones with intermediate to low values, which are respectively highlighted in yellow and green.

By comparing Figures 1 and 2, it is possible to observe the proximal incident areas. In Figure 1, they are represented by the higher density of cases per km^2^ and in Figure 2 by the census areas with the highest crude avoidable admission rates.

**Figure 3:**
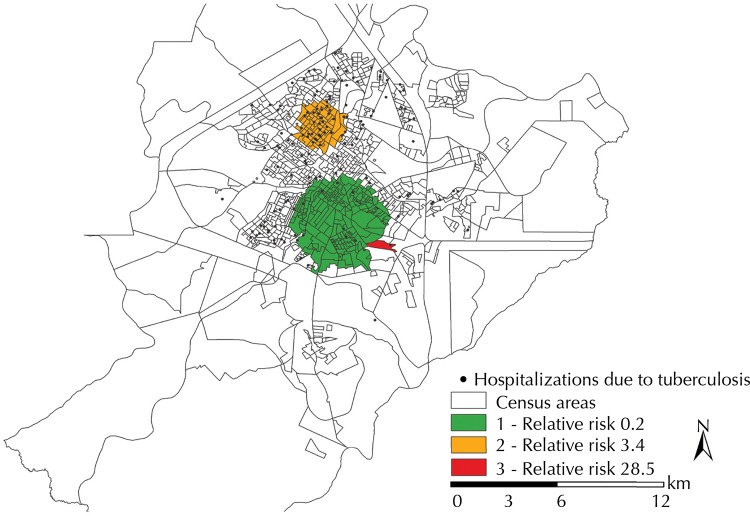
Spatial clusters of avoidable hospitalizations due to tuberculosis, as controlled by the populations of census areas and by sex and age. Ribeirao Preto, SP, Southeastern Brazil, 2006-2012.

In the purely spatial scan statistic, three statistically significant clusters were identified (Figure 3). The first two were considered of high risk and the third one was considered a protection factor.

Cluster 1 (p = 0.015), highlighted in green, comprised 214 areas, a population of 103,903 inhabitants, eight hospitalization cases, an average rate of 1.1 cases per 100,000 inhabitants/year, and RR of 0.2 (95%CI 0.2–0.3), being characterized as an area of protection for avoidable hospitalizations. Concerning the space time scan statistic, no statistically significant clusters were found, considering the period from 2006 to 2012.

Cluster 2 (p = 0.001), highlighted in orange, comprised 70 census areas, a population of 49,264 inhabitants, 38 hospitalization cases with an average rate of 10.9 cases per 100,000 inhabitants/year, and RR of 3.4 (95%CI 2.7–4.4).

Cluster 3 (p = 0.001), highlighted in red, only comprised one census area, a population of 804 inhabitants, six hospitalization cases, an average rate of 104.8 cases per 100,000 inhabitants/year, and RR of 28.5 (95%CI 22.4–36.6). It should be pointed out that the municipality’s Provisional Prison Center is located in this area.

## DISCUSSION

The theme maps obtained in the spatial scan analyses are relevant tools in evaluating the risk of avoidable hospitalizations due to tuberculosis, as they allow visualizing the spatial distribution of an event, identifying areas of higher risk and more in need of improvement regarding health surveillance and disease control measures.

Regarding the profiles of hospitalized individuals, these were predominantly males of ages between 15 and 64 years, with pulmonary tuberculosis as its clinical form. Studies^22,24^ conducted in other Brazilian cities also found a higher prevalence of tuberculosis among men, who were found to have 1.6 more chances of being admitted to hospitals than women. Regarding their ages, the more frequently afflicted people were the economically active ones, which especially jeopardizes the economy in developing countries, which are the most affected by tuberculosis, as corroborated by the literature^3,8,f^.

The large number of tuberculosis cases in people aged above 64 years corroborates the literature, which suggests the disease starts tending to happen in older groups. This shift may be the result from the efficiency in BCG vaccination and the population growing older^5^. In turn, the occurrence of admission of children (minimum age of six years) indicates the diagnostic delay in adult patients and flaws in the surveillance system of index cases and contact control^29^.

In the analyzed period, despite 84.0% of the patients having been discharged, it was not possible to verify the final outcome for the treatment of tuberculosis, an important issue which was also missing in another study conducted in Rio de Janeiro^22^. To solve it, HIS-SUS would have to be integrated to other information systems such as TB-WEB. Such integration could promote a more global understanding of the health statuses of these patients when they return to their communities. Such understanding, in turn, could allow coordinating care and planning more effective measures to control the disease that have positive healing results and treatment compliance.

Regarding the clinical forms of the disease, among inpatients, pulmonary tuberculosis predominated, and that corroborates the literature^22^. However, an important share of patients was not mentioned to have confirmed bacteriological or histological results (ICD 16.2). Such situation suggests the fragile conditions of health care service systems upon requesting sputum bacilloscopy exams, the most cost-effective method to diagnose tuberculosis.

Despite diagnosing tuberculosis being relatively simple, both in outpatient and inpatient situations, coughing that lasts for over three weeks has been neglected as a symptom of the disease, which leads to delayed diagnosis and choosing inappropriate or expensive methods, which are hard to afford for a health care system of limited resources. In hospital environments, sputum examination has not been the method of choice when tuberculosis is suspected, which is related to the institutional culture that favors more cutting-edge technological methods^2^.

Another issue regarding the findings is the lack of active initiatives in the community, such as searching cases of patients who cannot have access to the services. That would lead to early community diagnoses, and lower costs and numbers of admissions^25^.

Consequently, hospitalizations are found to be sensitive indicators to the solving ability and effectiveness of health care systems, and they are useful to monitor the PHC quality of a certain territory^1^. Nonetheless, the initiatives conducted by PHC services are not egalitarian, which helps clusters forms, as shown by the Kernel method and confirmed by the space scan statistic. The clusters located in this study are located in the northern and western districts, which correspond to the areas of highest social vulnerability^4,g^ and higher prevalence of tuberculosis, as pointed out in a study by Hino et al.^14^ (2011) and Roza et al.^27^ (2012). The northern zone has the worst social indicators among the five health care districts and the highest number of people per household, being host to the highest number of subnormal clusters in the city, reason why it demands more efficient action and services concerning public policies and sector management. The western zone, in turn, has the most complex network of services in terms of technological density, the second highest percentage of users exclusively from the Brazilian Unified Health System (SUS), a considerable number of inhabitants per household with the highest percentage of children and adolescents, and predominance of intermediate social classes, with monthly income ranging from one to five-fold the established minimum monthly wage^g^.

Although the districts have differences regarding social vulnerability, PHC has a determining role in social protection and equitableness^h^. In this logic, the services must be supplied according to the demand for them, both in terms of quantity and quality^30^. PHC must be stronger, more comprehensive, and capable of promoting the sector management for widening the access of users in territories with less social opportunities^18^.

Nevertheless, this will only be possible when PHC services have qualified staff, with support and logistic systems^19^. It is also important for both the population and the workers and managers to accumulate culture competency^30^, to recognize the function of PHC and its organization logics.

In the area analysis, the map only with crude estimates may generate wrong conclusions. It hinders the interpretation, due to their high instability to measure the risk of a certain event, especially when the population in the affected region is small^19^, as is the case of the census areas. Thus, after the smoothing, despite spatial approaches showing similar results, the number of areas with a zero coefficient was observed to decrease, as their estimate rates incorporate the ones of their neighbors.

The scan analysis allowed stratifying the municipality in risk areas, which enabled visualizing spatial clusters that were capable of outlining homogeneous areas, measuring the risk of avoidable hospitalizations due to tuberculosis, thus minimizing the possible damage to the population exposed^7^. Among the three clusters found in the study, two were found to have a high risk. The cluster of highest risk is located in the municipality’s Provisional Prison Center, where prisoners live in social vulnerability conditions, in a crowded, inadequate physical structure.

These areas are important niches for the dissemination of tuberculosis and the disease prevalence among prison communities, and up to 50 times as high as the one in the general population^24^. Health care sub-systems operating inside prison units are still rarely connected to PHC services. Thus, it is important to improve the constitution of networks and the definition of common responsibilities and goals^24^. The community that navigates through these spaces is under epidemiological risk; therefore, cooperative strategies must be created to break the transmission chain of the disease.

Concerning the non-identification of space-time clusters, the distribution of avoidable hospitalizations due to tuberculosis was found to be consistent throughout time. Maybe a longer period may result in differences being observed.

The use of ecological studies does not allow inferring the municipality findings for the individual level^20^. Another limitation in this study refers to the use of HIS-SUS, which only contains data regarding admissions to public hospitals. However, a great obstacle health care systems face nowadays is the difficult exchange of information between the private sector and the government, which demands mechanisms that enable fluid communication between them, thus allowing the state to also be aware of health conditions that required hospitalization in the private sector.

The areas identified as being of protection must be carefully analyzed, as they may be spaces where cases are not always notified. Another limitation to be pointed out regards to the use of secondary data, which may have given findings some bias for not being complete.

In this study, it was not possible to check whether patients were admitted again, or to assess the average length of each hospitalization. It would be interesting to complement these variables in future studies, and to observe these patients’ transference of care to PHC systems or to reference centers, and to follow up these cases.

Recognizing the risk areas may supply subsidies to the conduction of health care initiatives in an equitable, fair way, thus enabling the choosing of priority groups in terms of supply and initiatives of health care services^18^.

Stratifying the risk areas must be the basis for the situational strategic planning; therefore, our study may indicate processes concerning local health care system management. Not only has it allowed observing the municipality as a whole, but also as a collection of very heterogeneous services, which comes to show the importance of governance in local systems and of defining core values and mission and vision statements that are unique among the services.

Hospitalizations due to tuberculosis reflect its delayed diagnosis, which brings physical, psychological, and social consequences for patients and their families. In terms of management, it is a costly event for public health care systems, which have limited budgets.

## Footnotes

a World Health Organization. Global tuberculosis report 2014. Geneva; 2014 [cited 2014 May 10]. Available from: http://apps.who.int/iris/bitstream/10665/ 137094/1/9789241564809_eng.pdf?ua=1

b Ministério da Saúde, Secretaria de Atenção à Saúde. Portaria nº 221, de 17 de abril de 2008. Define a Lista Brasileira de Internações por Condições Sensíveis à Atenção Primária. *Diario Oficial Uniao*. 18 abr 2008; Poder Executivo:70.

c Fundação Sistema Estadual de Análise de Dados. Informações dos municípios paulistas IMP, 2014. São Paulo (SP); 2014 [cited 2014 May 10]. Available from: http://www.imp.seade.gov.br/frontend/

d Centro de Vigilância Epidemiológica “Alexandre Vranjac”, Divisão de Controle da Tuberculose. Tuberculose. São Paulo; 2014 [cited 2014 May 10]. Available from: http://www.cve.saude.sp.gov.br

e Software for the spatial temporal and space-time scan statistics. Boston; 2005 [cited 2014 Oct 1]. Available from: http://www.satscan.org

f World Health Organization. The global plan to stop TB 2011-2015: transforming the fight towards elimination of tuberculosis – reprinted with changes, 2011. Geneva; c2010 [cited 2015 Sep 10]. Available from: http://apps.who.int/iris/ bitstream/10665/44437/ 1/9789241500340_eng.pdf

g Ribeirão Preto. Secretaria Municipal de Saúde, Departamento de Vigilância em Saúde e Planejamento. Fatores de risco relacionados à saúde da população residente na zona urbana de Ribeirão Preto (SP) 2008-2011. Ribeirão Preto (SP) [cited 2014 Sep 26]. Available from: http://www.ribeiraopreto.sp.gov.br/ssaude/vigilancia/vigep/fatores-risco.pdf

h Pan American Health Organization. Renewing primary health care in the Americas: a position paper of the Pan American Health Organization/World Health Organization (PAHO/WHO). Washington (DC); 2007 [cited 2015 Sep 10]. Available from: http://apps.who.int/medicinedocs/documents/s19055en/s19055en.pdf
